# Duplication of the Plantaris Tendon and Its Clinical Significance: A Case Report

**DOI:** 10.7759/cureus.75493

**Published:** 2024-12-10

**Authors:** José Araújo, Nuno Pais, António Andrade

**Affiliations:** 1 Orthopedics and Traumatology, Unidade Local de Saúde do Nordeste, Macedo de Cavaleiros, PRT; 2 Orthopedics and Sports Medicine, Clínica Espregueira-Mendes, Fédération Internationale de Football Association (FIFA) Medical Center of Excellence, Porto, PRT

**Keywords:** achilles, duplication, plantaris, tendinopathy, tendon

## Abstract

The plantaris tendon may be absent in some individuals, indicating its unclear function. Anatomically, the plantaris tendon originates from the lateral femoral condyle and has a variable course and insertion point at the calcaneal tuberosity. The plantaris tendon may influence conditions such as Achilles tendinopathy, particularly in its midportion, whether by its close relation to the calcaneal tendon or adhesions between both tendons. This case study involves a 38-year-old male patient with an Achilles tendon rupture; two plantaris tendons were discovered during surgical exploration. An anatomical variation was described before in a cadaveric study, where two plantaris muscles were encountered during specimen dissection, and the accessory tendon was inserted in the crural fascia proximal to the calcaneal tendon. The relationship between anatomical variations of the plantaris tendon and Achilles tendon injuries remains an area of ongoing research, and current findings suggest that while there may be a correlation, definitive conclusions cannot be drawn. This case reports a rare anatomical variation encountered during routine surgery, and despite not establishing a relation to tendon rupture, it can alert other surgeons to the anatomic variability of the plantar tendon and its possible role in the treatment of Achilles tendinopathy.

## Introduction

The plantaris tendon may be absent in some individuals, up to 7-20%, according to some authors [1-2], and others believe its function is questionable [3]. The plantaris muscle is biarticular and acts together with the gastrocnemius, but it is believed to be less significant as either a flexor of the knee or a plantar flexor of the ankle with knee extension, and having more of a proprioceptive function for the larger, more powerful plantarflexors, as it contains a high density of muscle spindles [4]. It is often used as a graft in the repair of structures such as the Achilles tendon [5], ligaments of the ankle [6-7], or flexors of the fingers [8]. Its anatomy originates from the lateral femoral condyle, a small muscle belly posterior to the popliteal muscle and anterior to the lateral head of the gastrocnemius. It descends as a tendon toward the medial crural region, between the soleus and medial gastrocnemius [9]. Distally, the course and insertion are associated with high variability [10-11]. Independent insertion at the calcaneal tuberosity is frequently present, whether as a bony attachment or with fascial adhesion to the flexor retinaculum. In an anatomical study, Olewnik et al. described five types of insertion into the calcaneal tuberosity based on its shape, relation to the calcaneal tendon, and the exact insertion point in the calcaneal tuberosity [12]. The anatomy is particularly relevant, as some authors believe that it may influence hindfoot disturbances such as Achilles tendinopathy, most commonly in its mid-portion (where firm connections between the plantaris and calcaneal tendon were found in a cadaveric study) in running and jumping athletes, but is also seen in less active middle-aged patients [13]. Steenstra and van Dijk observed situations in which the plantaris, Achilles tendons, and the paratenon were closely associated and accompanied by an inflammatory process [14]. Annamalai et al. described an anatomical variation in a cadaveric dissection in which two plantaris tendons were present and inserted with multiple attachments to the crural fascia [15]. We describe a case report in which we found a rare anatomical variation: a duplicated plantaris tendon during standard Achilles tendon repair surgery.

## Case presentation

**Figure 1:**
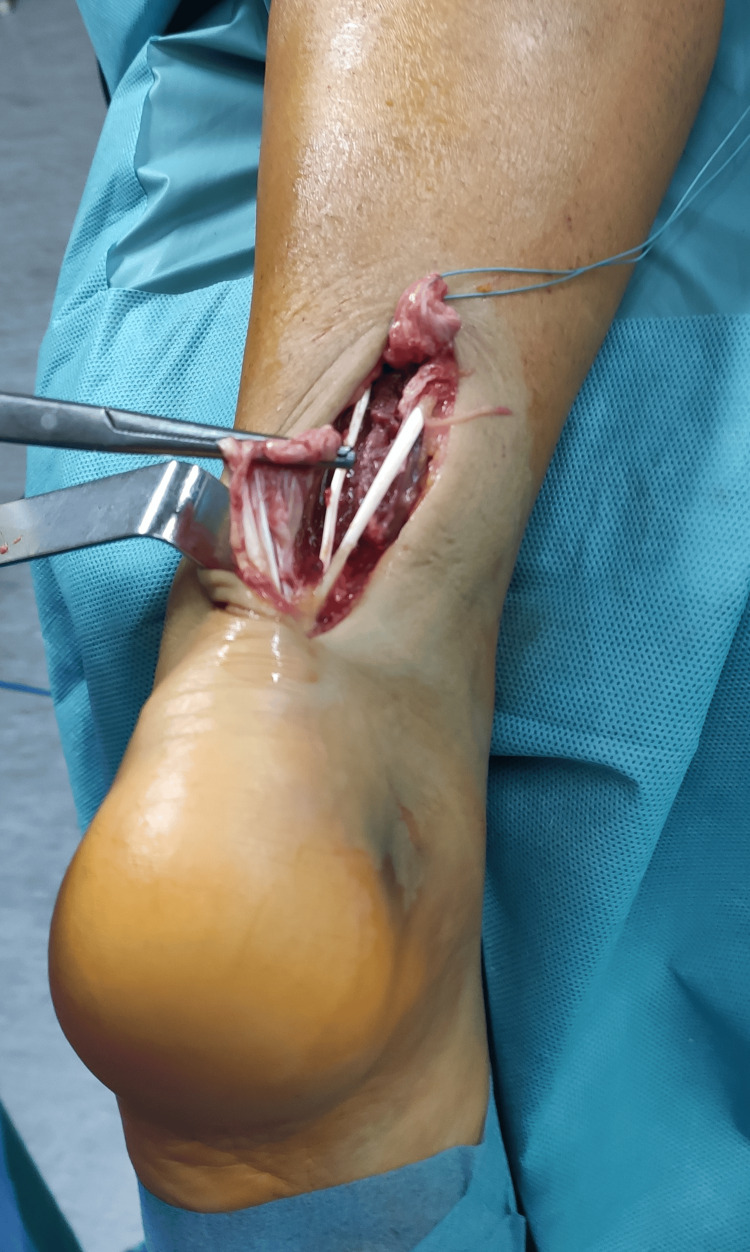
Achilles tendon rupture with underlying layer

**Figure 2:**
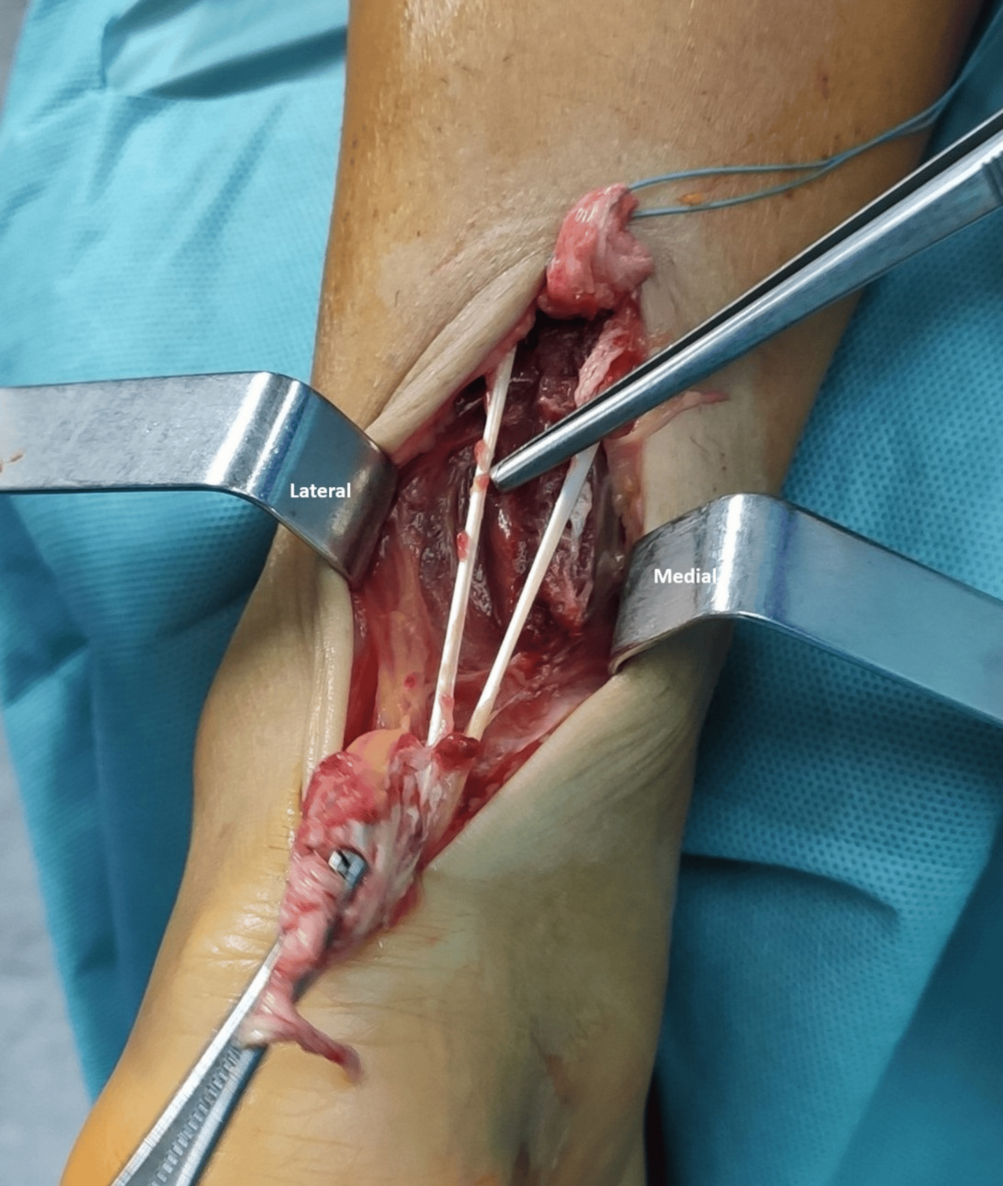
Duplicated plantaris tendon with apparent common distal trajectory

A healthy 38-year-old male presented with an injury to the left ankle, clinically with absent plantar flexion, a positive Thompson’s test, and a palpable gap 4 cm proximal to the calcaneal tuberosity. Ultrasound imaging confirmed the diagnosis of traumatic Achilles tendon rupture. No other injuries or any anatomic variations were found preoperatively. After the initial workup, the patient was admitted to surgery eight hours after the trauma. The initial surgical assessment focused on exploration and irrigation to assess the damaged Achilles tendon. Deep exploration of the crural region revealed the presence of two plantaris tendons (Figures 1-2). We could not access the proximal and distal trajectories through our dissection. The Achilles tendon reconstruction was accomplished with open “end-to-end” repair, and after closure, a splint with a slight equinus was applied. After four weeks, the splint was removed, and the patient initiated mobilization with partial weight bearing. He went on full recovery after four months. No complications were observed during follow-up. The patient went on a loss of follow-up and did not undergo a subsequent imaging study, therefore not accessing the proximal and distal course of the duplicated plantaris.

## Discussion

Several studies report intrasubstance degeneration of the Achilles tendon in acute rupture, suggesting that tendinopathy may precede tendon rupture. Furthermore, Olewnik et al. reported anatomical variations of the plantaris tendon might affect and predispose midportion tendinopathy [12]. As described before, only one article was found when searching for literature describing duplication of the plantaris tendon. However, according to Herzog, an accessory plantaris muscle belly was found at the knee level in 6.3% of 1000 patients [16]. Although no meta-analyses were performed, no articles were found regarding a duplicated plantaris tendon's relation to tendon rupture. To our knowledge, no anatomical variations like the one described were found during routine surgery. We hypothesize that, in relation to literature, this anatomical variation may correlate with rupture, but no conclusions can be found due to a lack of scientific data. The patient reported no pain before the rupture and had no clinical symptoms suggesting Achilles tendinopathy. Also, no risk factors, such as clinical conditions or chronic medications, were noted.

## Conclusions

Variability in the distal insertion of the plantaris tendon is frequently observed. This case reports a rare variation encountered accidentally during surgery, and no anatomical imaging was performed, therefore not accessing the relations between origin and insertion and comparison with the previous cadaveric studies. The relation between anatomic variations of the plantaris tendon and Achilles tendon rupture is still a topic of ongoing study and debate in the medical community, and further studies are needed to clarify the potential impact of the plantaris tendon on Achilles tendon injuries and to determine the specific factors that may increase the risk of rupture. Despite not establishing a relation to tendon rupture, this case can alert other surgeons to the anatomic variability of the plantaris tendon and its possible role in the treatment of Achilles tendinopathy.
